# Supplementary material for: Copper-on-nitride enhances the stable electrosynthesis of multi-carbon products from CO2
Source: Nat Commun. 2018 Sep 20;9:3828. doi: 10.1038/s41467-018-06311-0 (PMC6148248; doi:10.1038/s41467-018-06311-0)
Supplement: Supplementary file 1 — Supplementary Information [file 41467_2018_6311_MOESM1_ESM.pdf]

Copper-on-nitride enhances the stable electrosynthesis of multi-carbon products from CO_2

Zhi-Qin Liang,[1,2]† Tao-Tao Zhuang,[1]† Ali Seifitokaldani,[1]† Jun Li,[1,3] Chun-Wei Huang,[4] Chih-Shan Tan,[1] Yi Li,[5] Phil De Luna,[6] Cao Thang Dinh,[1] Yongfeng Hu,[7] Qunfeng Xiao,[7] Pei-Lun Hsieh,[8] Yuhang Wang,[1] Fengwang Li,[1] Rafael Quintero-Bermudez,[1] Yansong Zhou,[1] Peining Chen,[1] Yuanjie Pang,[1,3] Shen-Chuan Lo,[4] Lih-Juann Chen,[8] Hairen Tan,[1] Zheng Xu,[2] Suling Zhao,[2] David Sinton,[3] Edward H. Sargent[1*]

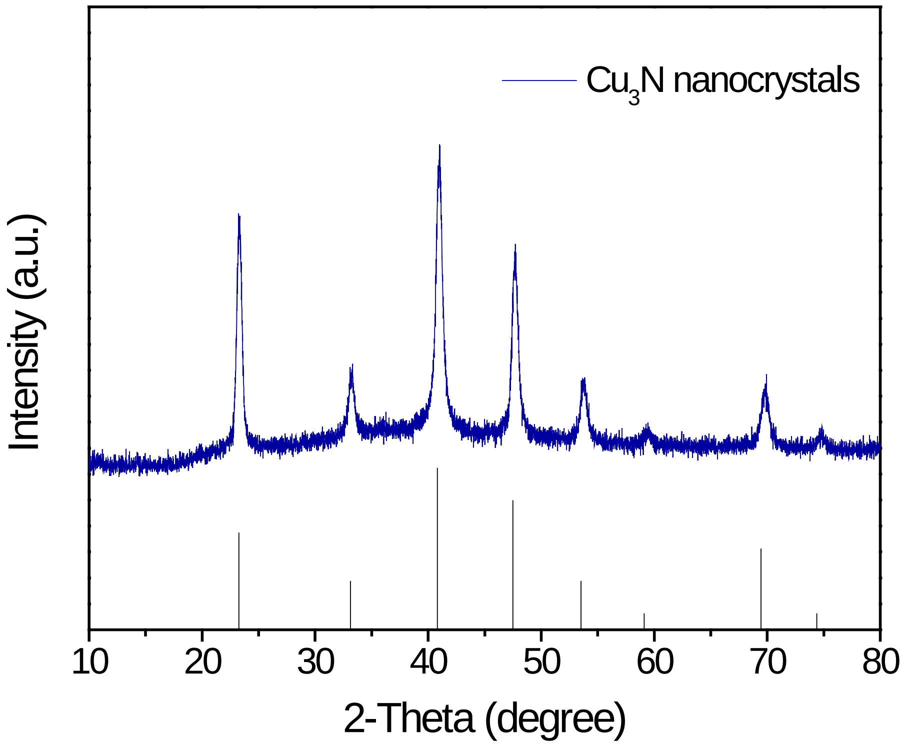

Supplementary Fig. 1. XRD pattern of Cu_3N nanocrystals.

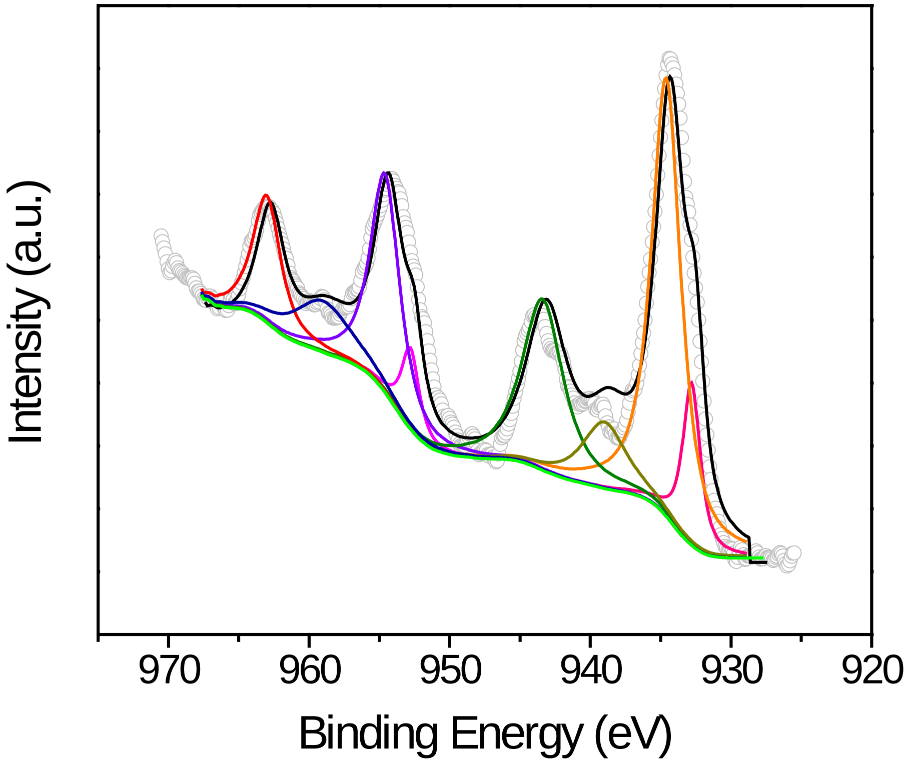

Supplementary Fig. 2. Cu XPS spectra of CuO-on-Cu_3N intermediates. The main peaks of Cu^{2+} $2p_{1/2}$, Cu^{+} $2p_{1/2}$, Cu^{2+} $2p_{3/2}$ and Cu^{+} $2p_{3/2}$ were located at 954.6 eV, 952.8 eV, 934.6 eV, and 932.8 eV, respectively. The corresponding satellite peaks are positioned at 963.1 eV, 959.3 eV, 943.5 eV, and 939.1 eV, respectively.

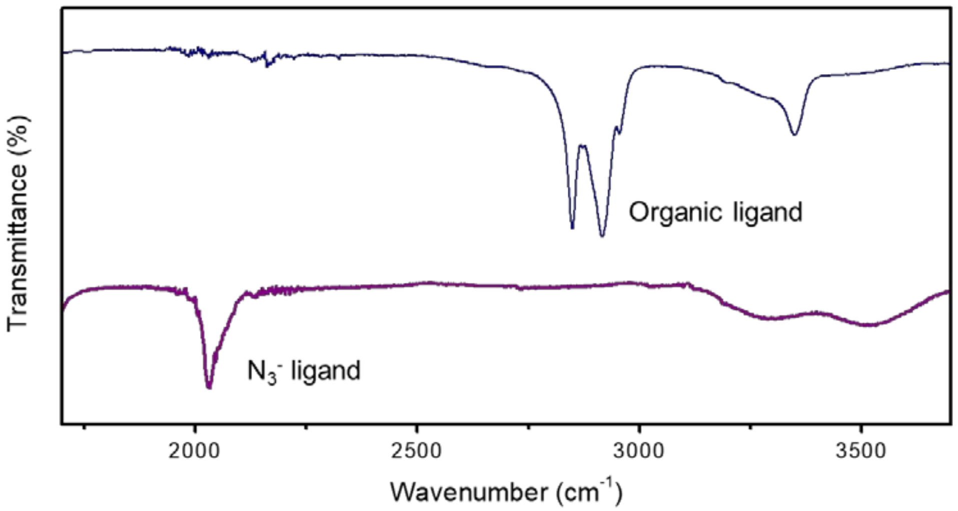

Supplementary Fig. 3. Comparison of FTIR spectra of Cu_3N nanocrystals with ODA ligands (blue line) and with N_3^- ligands (purple line).

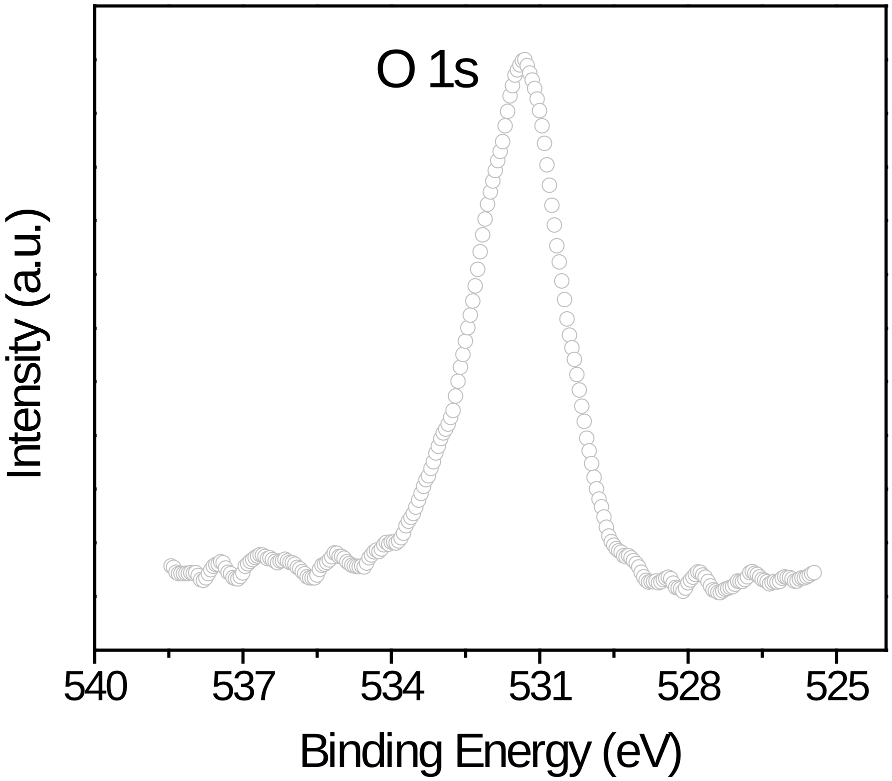

Supplementary Fig. 4. XPS spectrum of O 1s on CuO-on-Cu_3N intermediate.

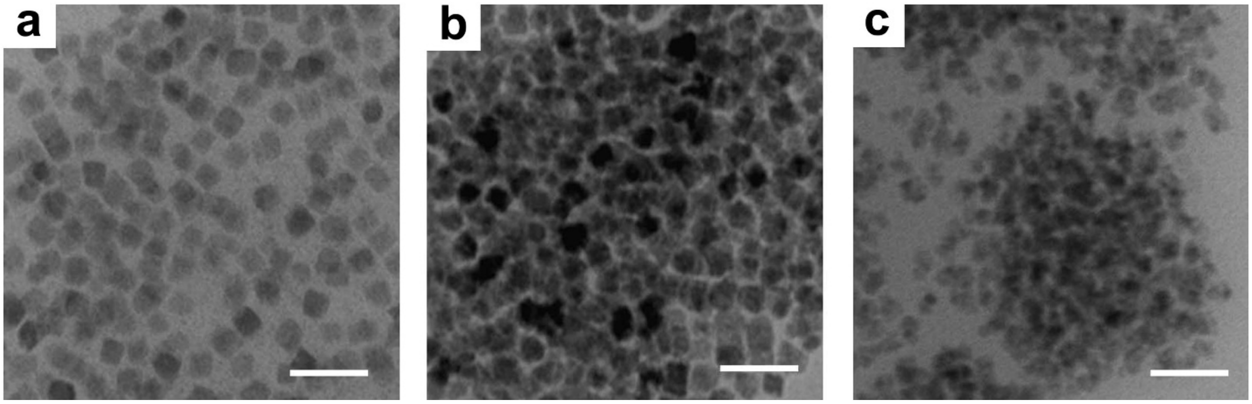

Supplementary Fig. 5. Low-resolution TEM images corresponding to designs steps of the electrocatalyst. a, Cu_3N nanocrystals with long organic ODA. **b,** Cu_3N nanocrystals with an oxide layer after N_3^- ligand exchange. **c,** Cu-on-Cu_3N nanocrystals after initial electroreduction. The scale bar is 100 nm.

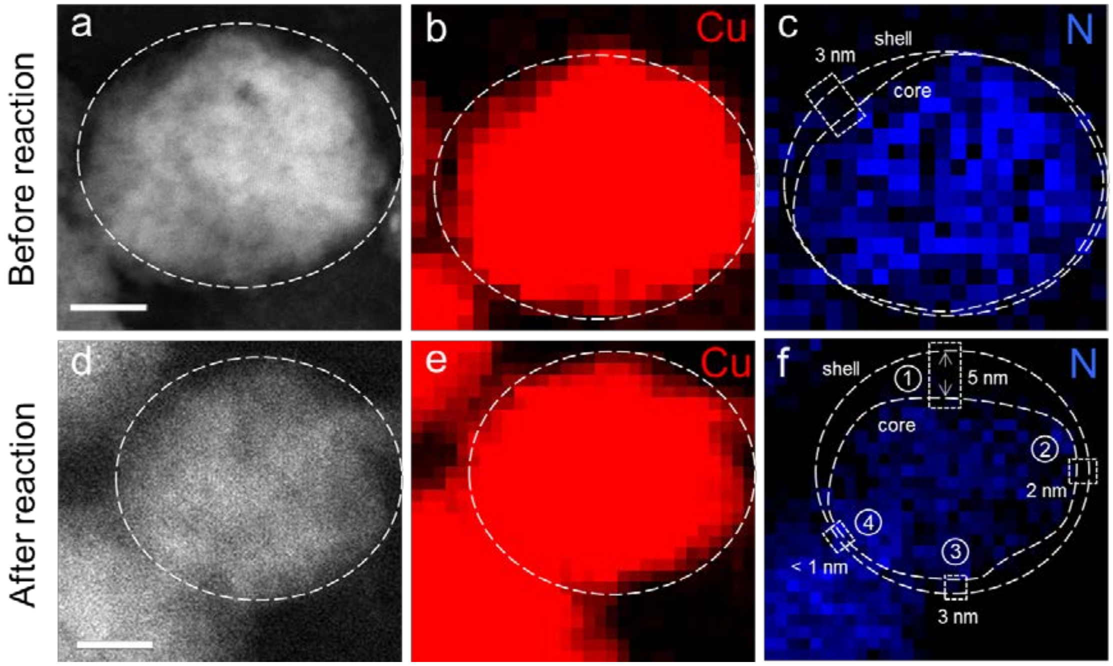

Supplementary Fig. 6. TEM characterization of the Cu-on-Cu_3N catalyst before and after two-hour reaction. **a** and **d,** HADDF-STEM images before (**a**) and after (**d**) reaction. **b-c**, STEM-EELS Cu and N element mapping of the particle in **a**. **e-f**, Cu and N element mapping of the particle in **d**. The scale bar is 10 nm.

Before reaction, the thickness of the surface Cu is below 1 nm in most areas (Supplementary Fig. 6c). After the two-hour reaction, the thickness is within 2 nm in the area 2 and area 4 (Supplementary Fig. 6f).

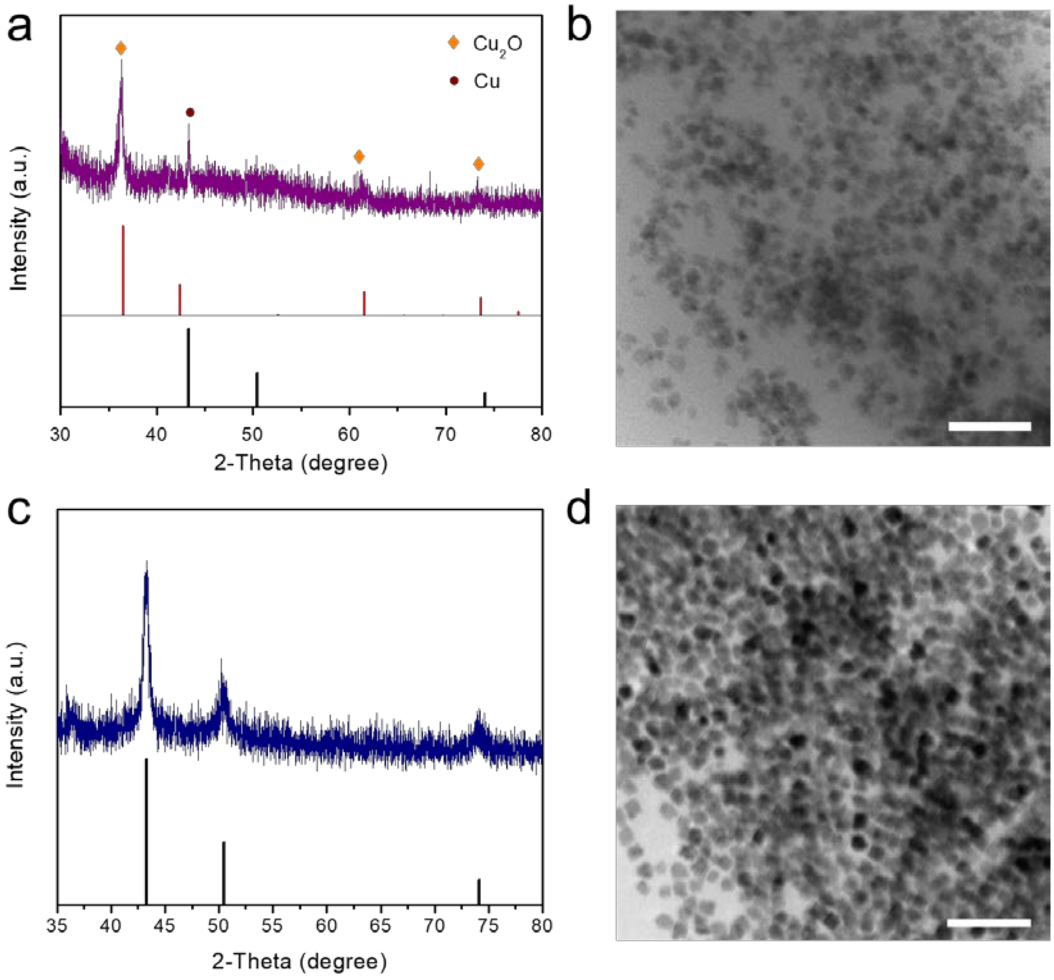

Supplementary Fig. 7. Characterization of Cu-on-Cu_2O and pure Cu catalysts. a and **b**, XRD pattern and TEM image of Cu-on-Cu_2O catalyst after initial electroreduction. **c** and **d,** XRD pattern and TEM image of pure Cu catalyst after initial electroreduction. The scale bar is 200 nm.

Cu-on-Cu_2O and pure Cu control catalysts were designed by three steps. First, Cu_2O and Cu nanocrystals were synthesized with ODA long organic molecules, respectively. The ODA long-chain ligand was then exchanged with the short-chain azide ligand. During this process, an oxide layer was formed outside the nanocrystals. Subsequently, the composite went through initial electroreduction, yielding Cu-on-Cu_2O and pure Cu catalysts, respectively.

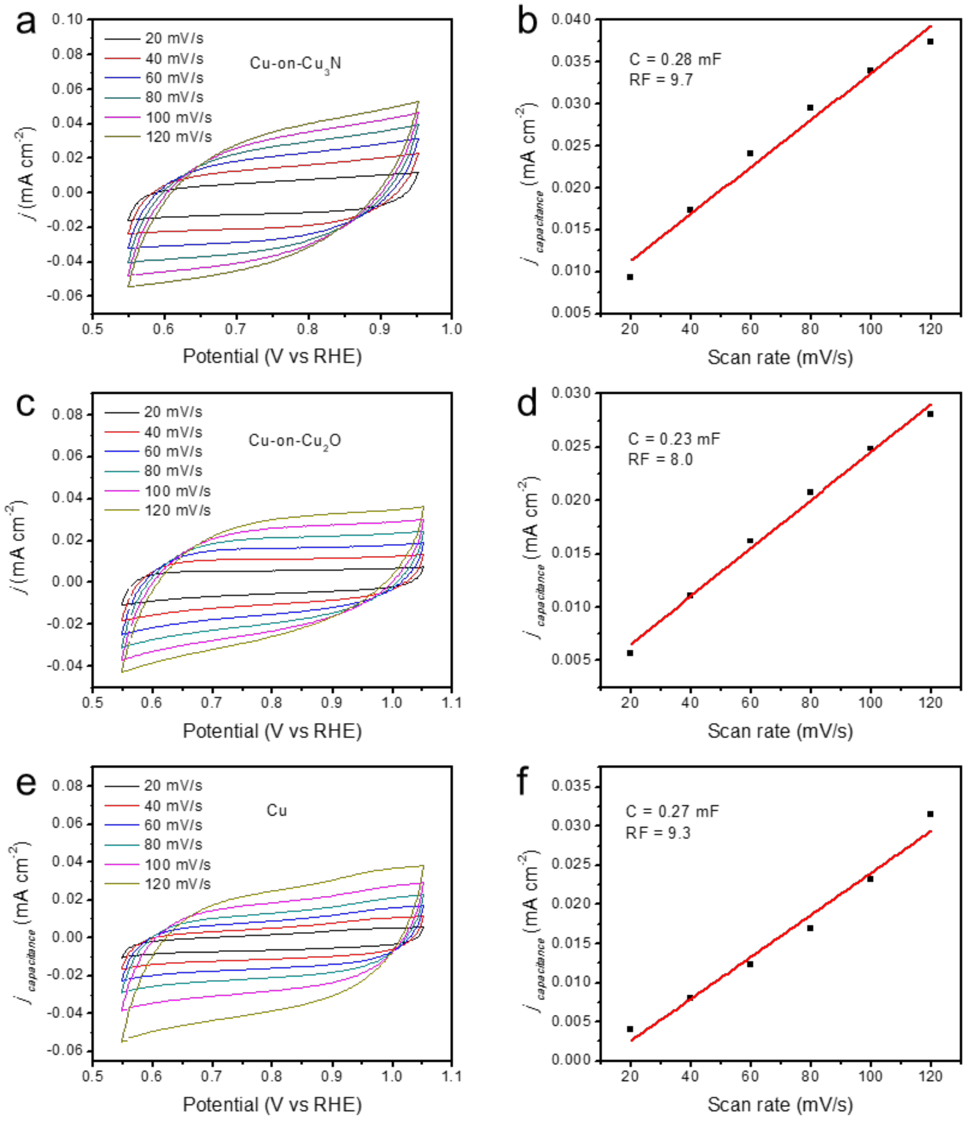

Supplementary Fig. 8. Electrochemical surface area (ECSA) measurements. a and **b,** Determination of double-layer capacitance over a range of scan rates on Cu-on-Cu_3N catalyst. **c** and **d,** Determination of double-layer capacitance over a range of scan rates on Cu-on-Cu_2O catalyst. **e** and **f,** Determination of double-layer capacitance over a range of scan rates on pure Cu catalyst.

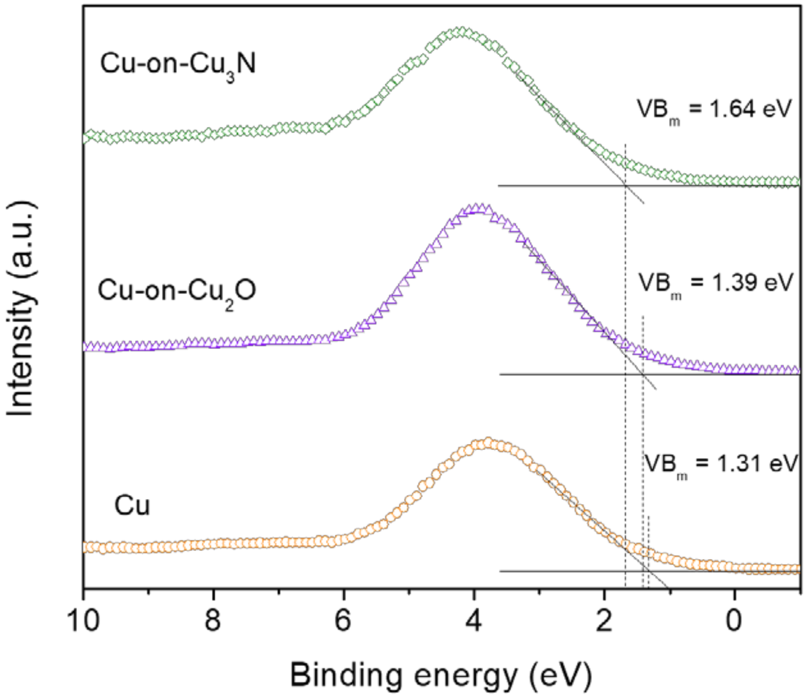

Supplementary Fig. 9. XPS valence band spectra at the surface on the three catalysts: Cu-on-Cu_3N catalyst (green dots); Cu-on-Cu_2O catalyst (purple dots); and pure Cu (orange dots).

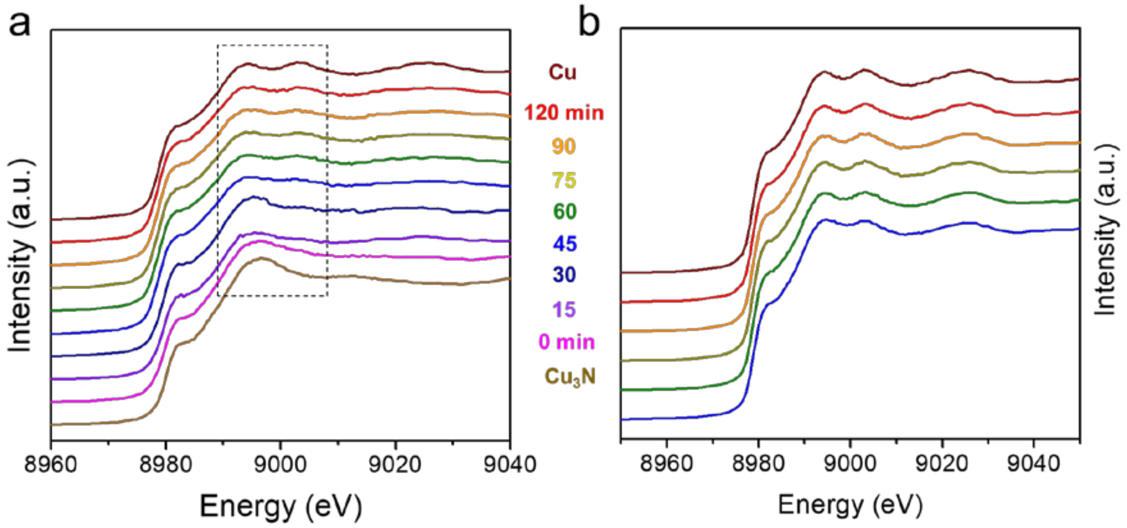

Supplementary Fig. 10. *In-situ* XANES spectra during CO_2 reduction. a, On Cu-on-Cu_2O catalyst, and **b,** On pure Cu catalyst.

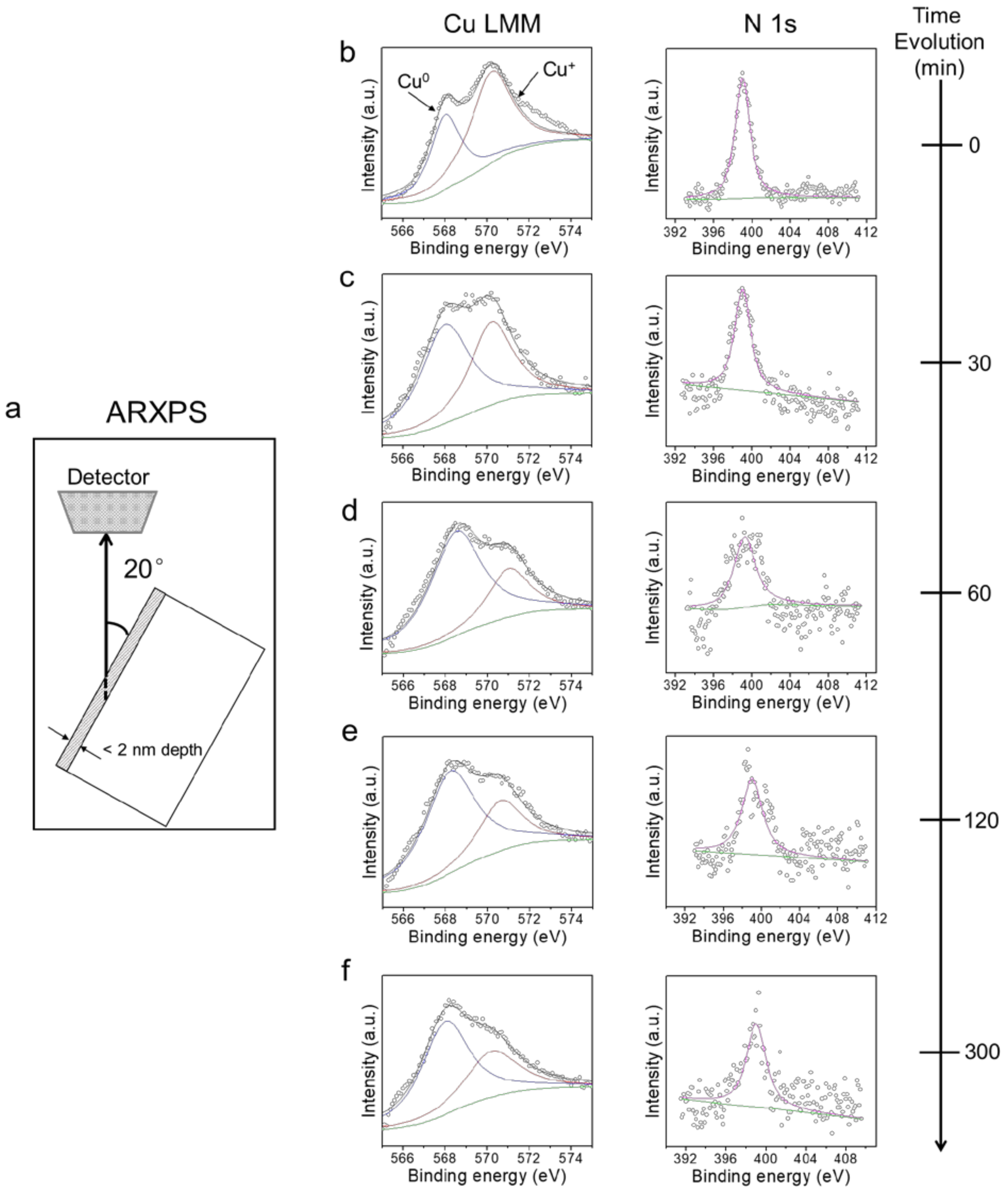

Supplementary Fig. 11. Angle-resolved XPS (ARXPS) characterization of the catalyst with time evolution under CO_2 reduction. a, Scheme of ARXPS analysis at 20° emission angle with respect to the sample surface. **b-f,** ARXPS results of Cu LMM (left column) and N 1s (right column) spectra obtained with different reaction time: **b,** 0 min, **c,** 30 min, **d,** 60 min, **e,** 120 min, and **f,** 300 min, respectively.

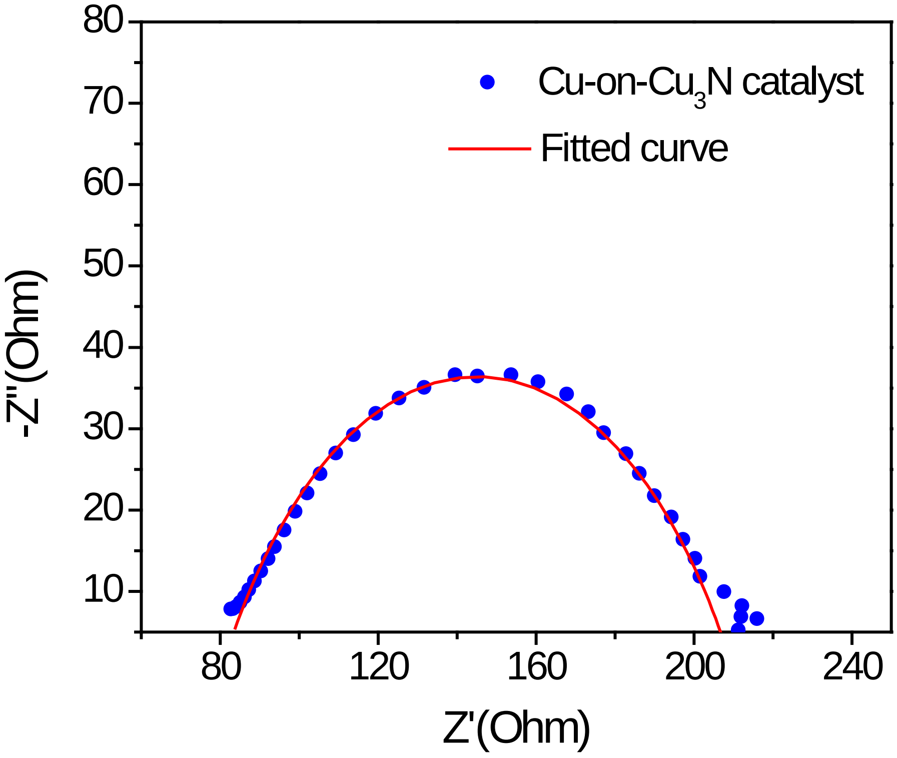

Supplementary Fig. 12. Electrochemical impedance spectroscopy (EIS) curve (blue dots) and its related fit result (red line) taken in the electrolysis cell with the Cu-on-Cu_3N working electrode in 0.1 M $KHCO_3$ electrolyte.

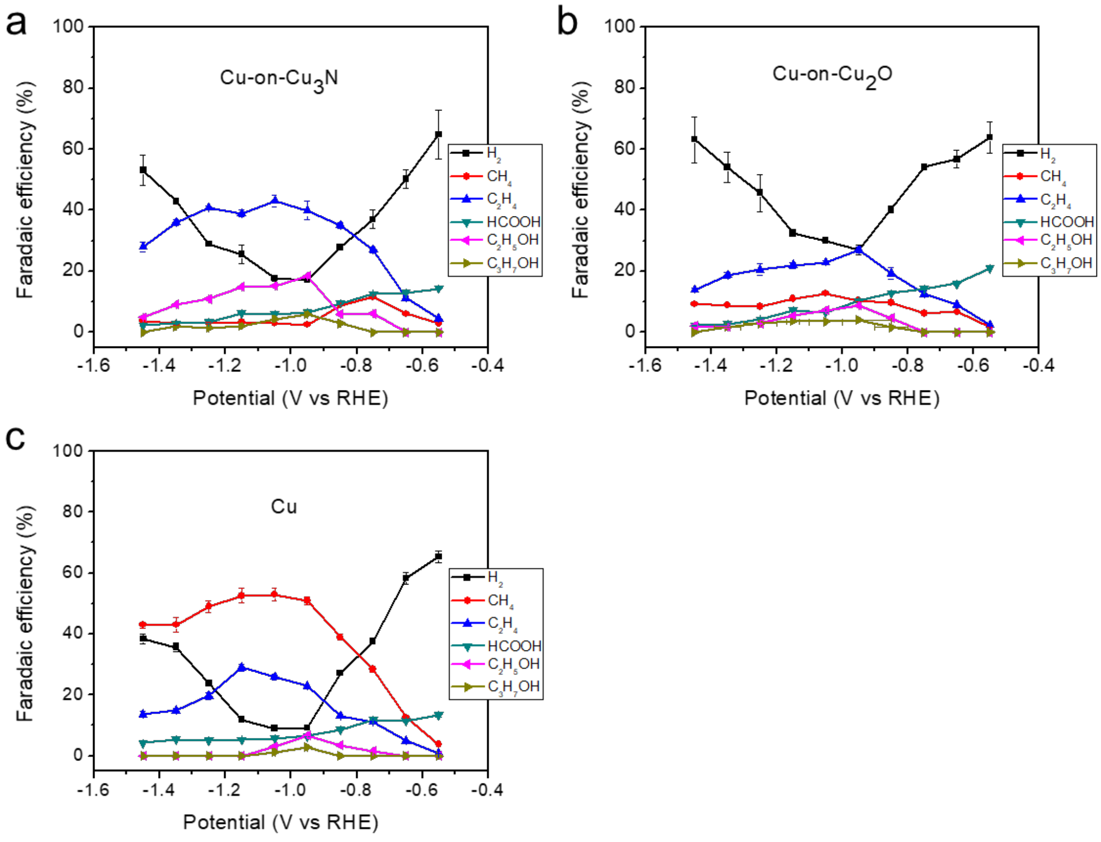

Supplementary Fig. 13. Product distribution of CO_2 reduction on the three catalysts. a, Cu-on-Cu_3N. **b,** Cu-on-Cu_2O. **c,** pure Cu. Experiments from **a** to **c** were performed in triplicates and the results are shown as mean ± standard deviation.

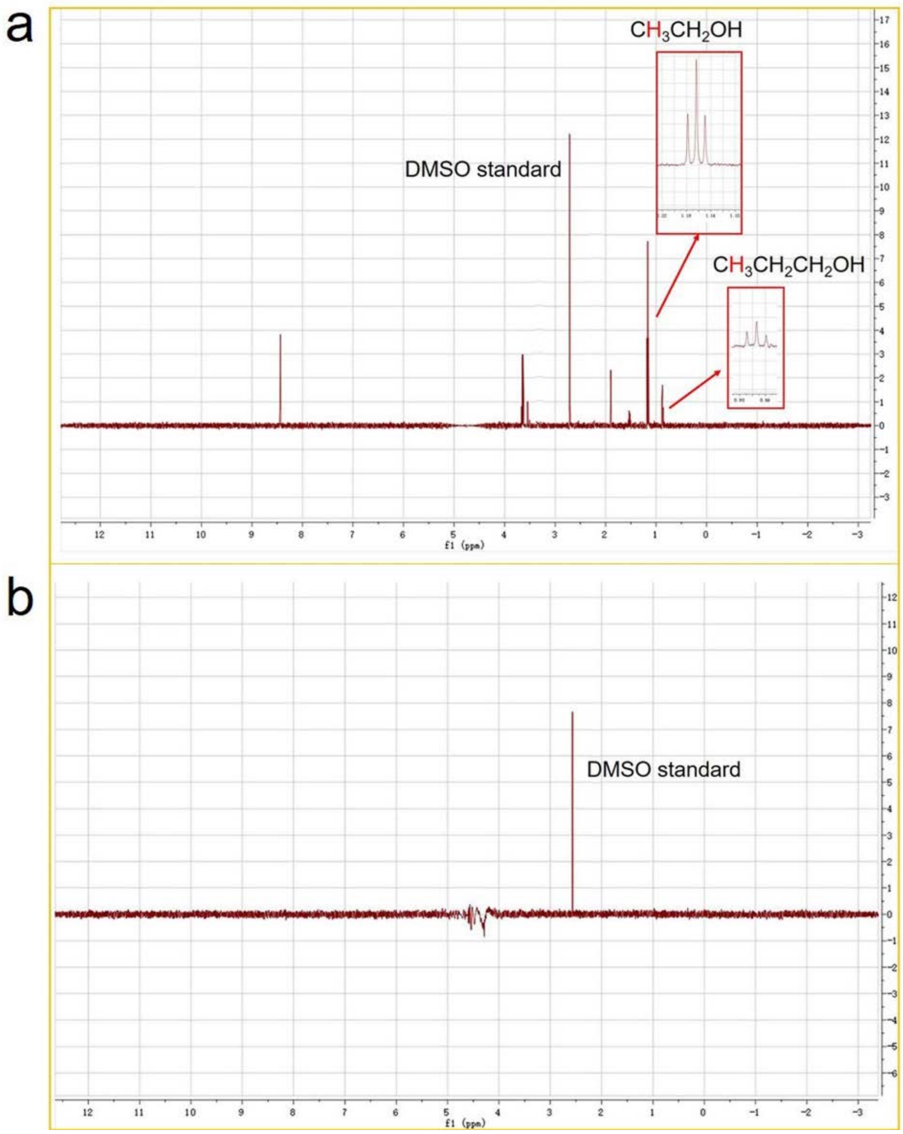

Supplementary Fig. 14. ^{1}H NMR spectra of CO_2 reduction products after reaction. a, In CO_2-saturated 0.1M $KHCO_3$ electrolyte. **b**, In N_2-saturated 0.1 M $KHCO_3$ electrolyte.

We ran the same samples in the N_2-saturated 0.1 M $KHCO_3$ solution as a control, and no product was detected besides H_2. We conclude that the products come from CO_2.

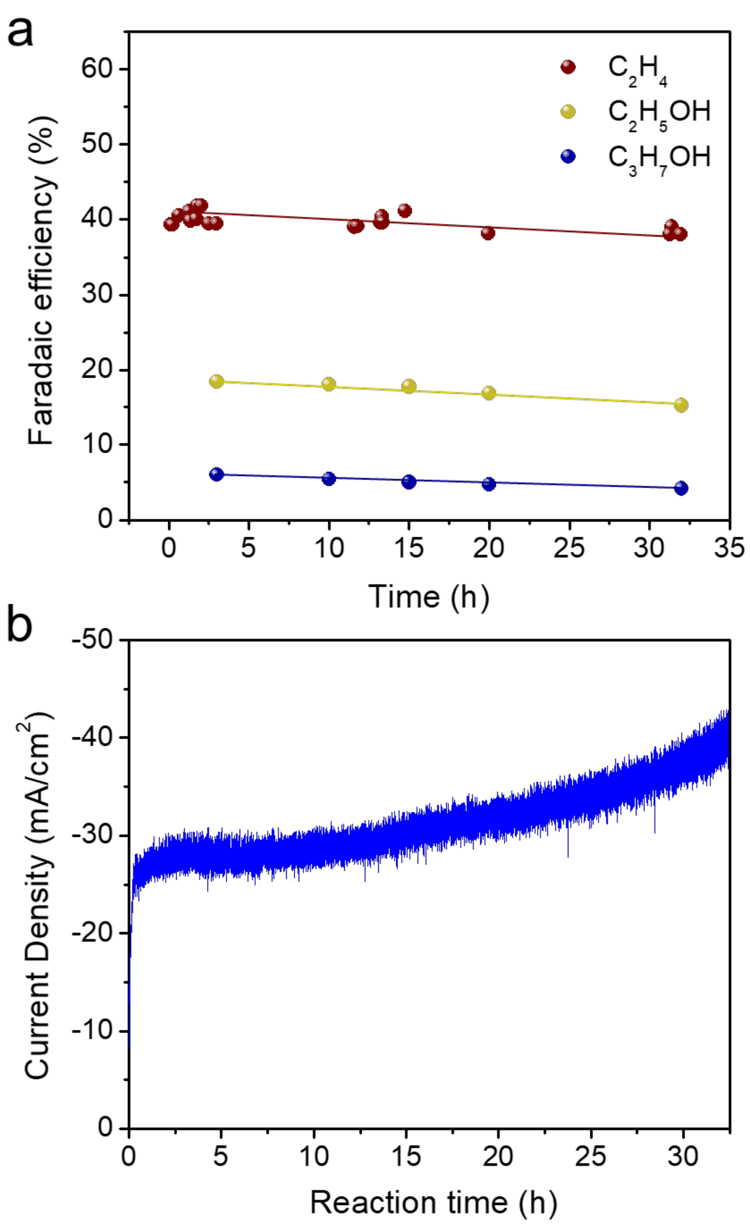

Supplementary Fig. 15. The stability of the Cu-on-Cu_3N catalyst. a, Faradaic efficiency of C_2H_4, C_2H_5OH, and C_3H_7OH during 32-hour CO_2 reduction. **b,** Chronoamperometry of the reaction operation using Cu-on-Cu_3N catalyst at -0.95 V vs RHE.

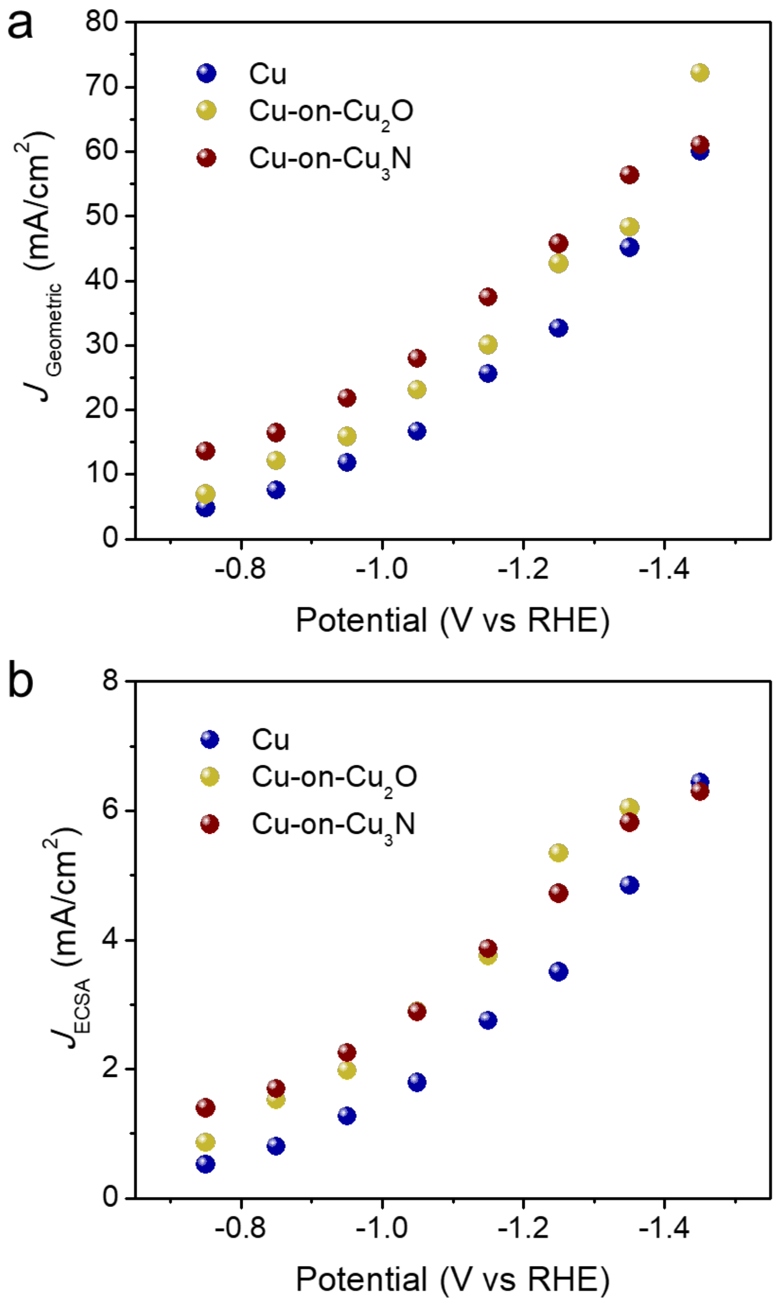

Supplementary Fig. 16. Current densities as function of cathode potentials on the three catalysts. a, Current densities are normalized on geometric area. **b,** Current densities are normalized on ECSA.

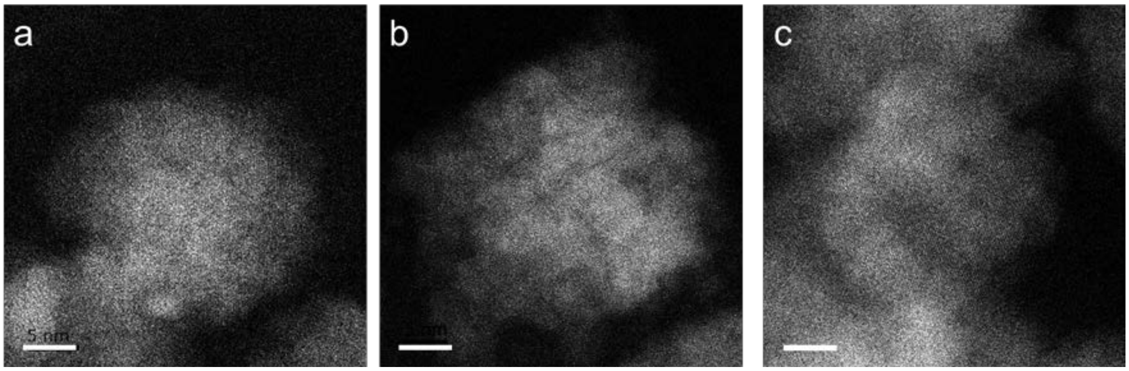

Supplementary Fig. 17. TEM images of the three catalysts after two-hour CO_2 reduction. a, Cu-on-Cu_3N. **b,** Cu-on-Cu_2O. **c,** pure Cu. The scale bar is 5 nm.

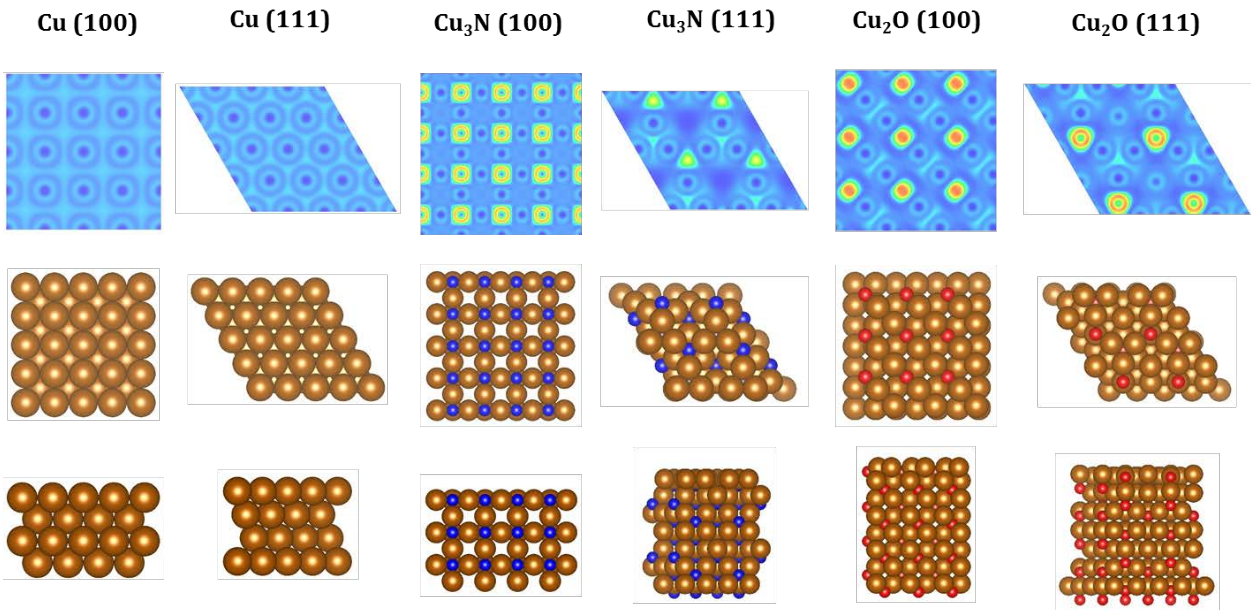

Supplementary Fig. 18. DFT models of the studied pristine crystals. Two (111) and (100) facets of copper (Cu), copper nitride (Cu_3N) and copper oxide (Cu_2O) are demonstrated here. Top panel shows the electron localization function (ELF); middle and bottom panels, respectively, show the top and side views of these structures.

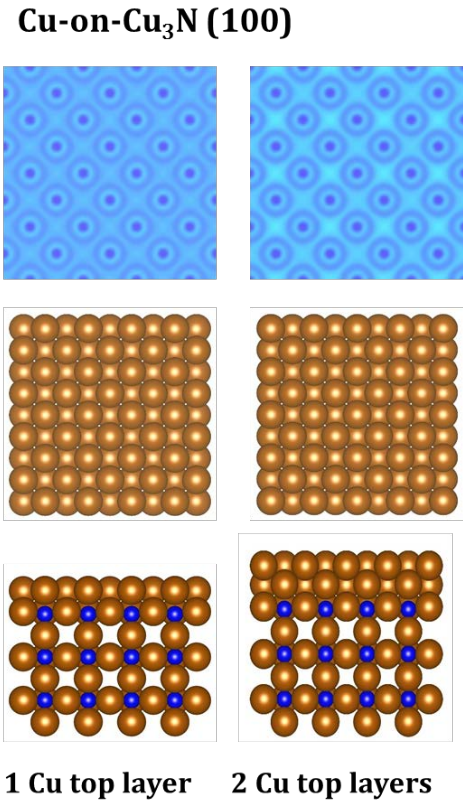

Supplementary Fig. 19. The DFT models of the studied Cu-on-Cu_3N crystals. Two different models of (100) facet with 1 and 2 layers of copper are demonstrated here. The top panel shows the electron localization function (ELF); the middle and bottom panels, respectively, show the top and side views of these structures.

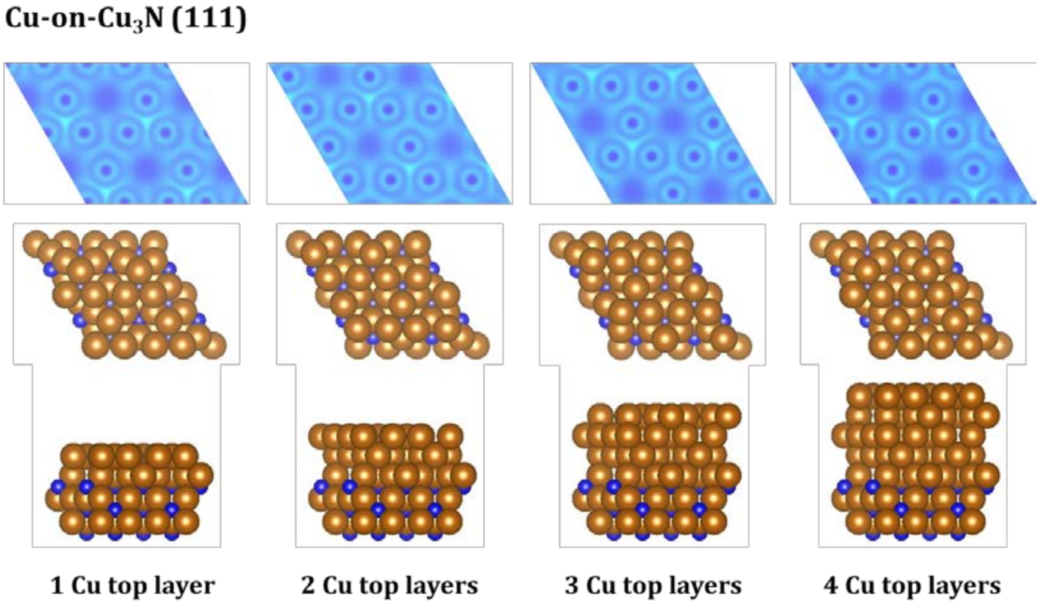

Supplementary Fig. 20. The DFT models of the studied Cu-on-Cu_3N crystals. Four different models of (111) facet with 1, 2, 3 and 4 layers of copper are demonstrated here. The top panel shows the electron localization function (ELF); the middle and bottom panels, respectively, show the top and side views of these structures.

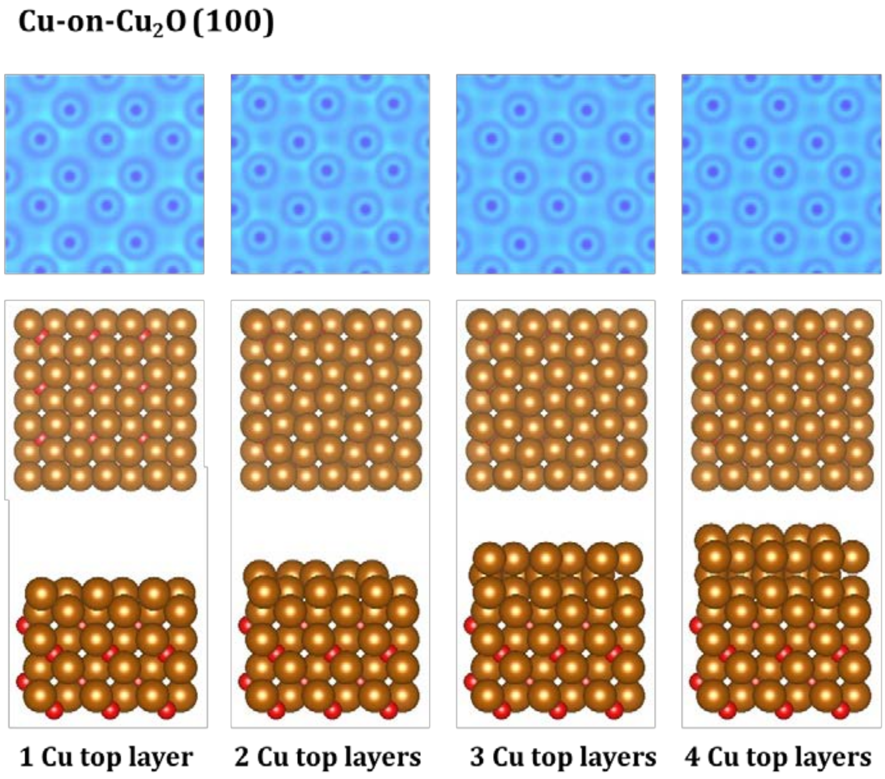

Supplementary Fig. 21. DFT models of the studied Cu-on-Cu_2O crystals. Four different models of (100) facet with 1, 2, 3, and 4 layers of copper are demonstrated here. The top panel shows the electron localization function (ELF); the middle and bottom panels, respectively, show the top and side views of these structures.

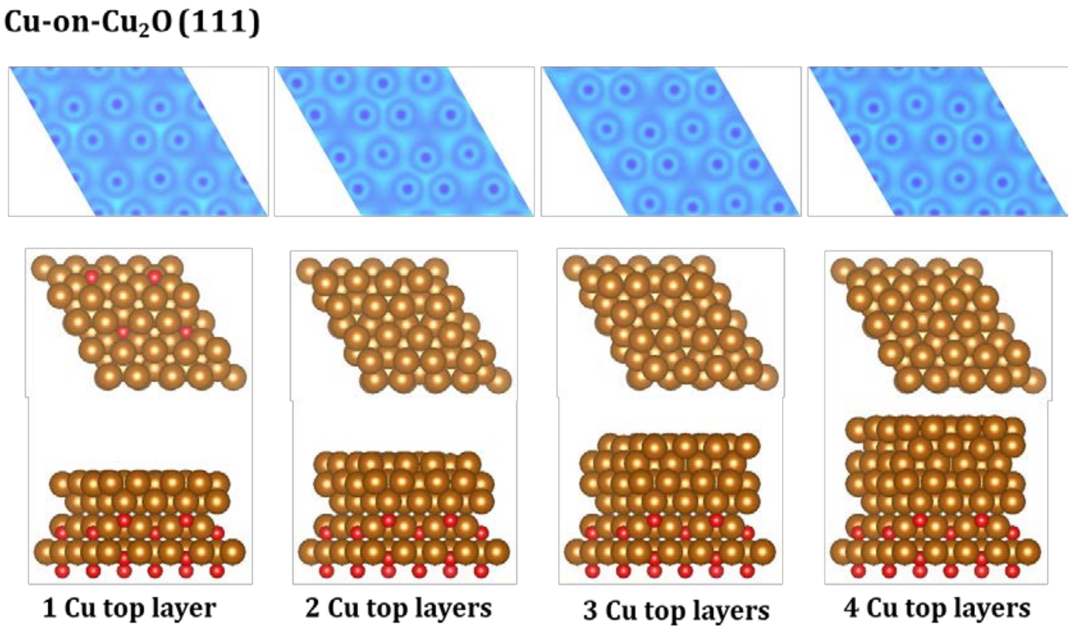

Supplementary Fig. 22. DFT models of the studied Cu-on-Cu_2O crystals. Four different models of (111) facet with 1, 2, 3, and 4 layers of copper are demonstrated here. The top panel shows the electron localization function (ELF); the middle and bottom panels, respectively, show the top and side views of these structures.

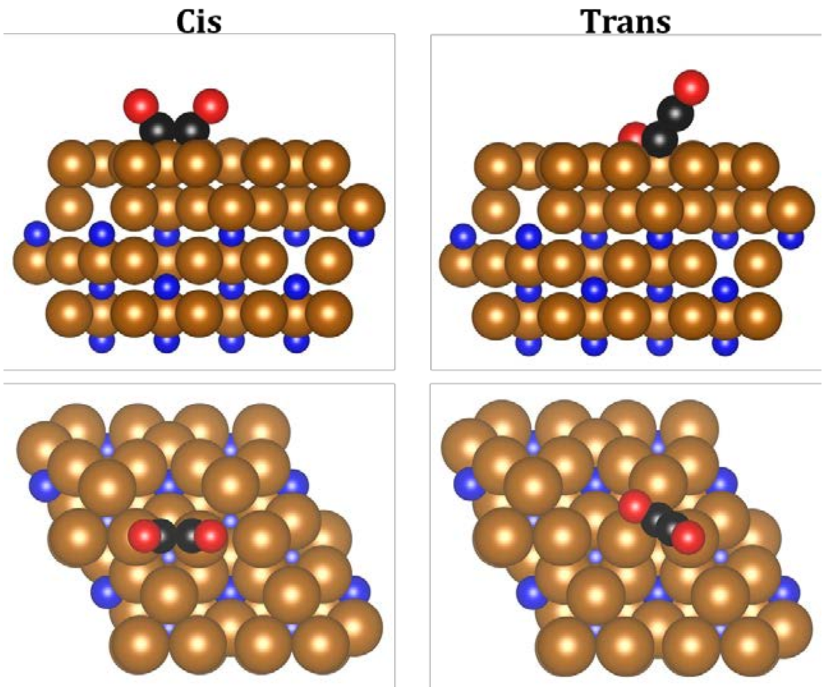

Supplementary Fig. 23. Initial configurations for CO dimerization. Two so-called “Cis” and “Trans” configurations considered for CO dimerization. Top panel shows the side view and the bottom panel shows the top view of these two configurations on (111) facet of Cu-on-Cu_3N model with 1 Cu top layer.

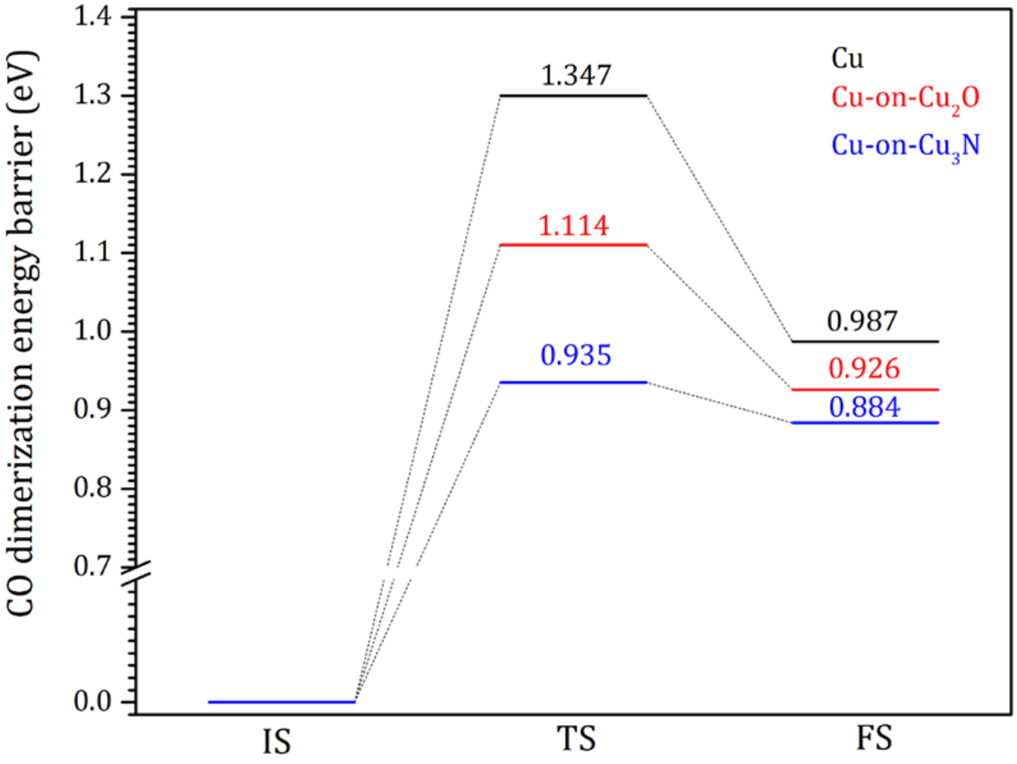

Supplementary Fig. 24. Activation energy barrier for CO dimerization. Thermodynamic and activation energy barriers (calculated by considering the transition state) for CO dimerization are demonstrated for (100) facet of Cu (black line), Cu-on-Cu_2O (red one) and Cu-on-Cu_3N (blue one). IS stands for initial state of two adsorbed CO, TS stands for the transition state, and FS stands for final state of OCCO intermediate.

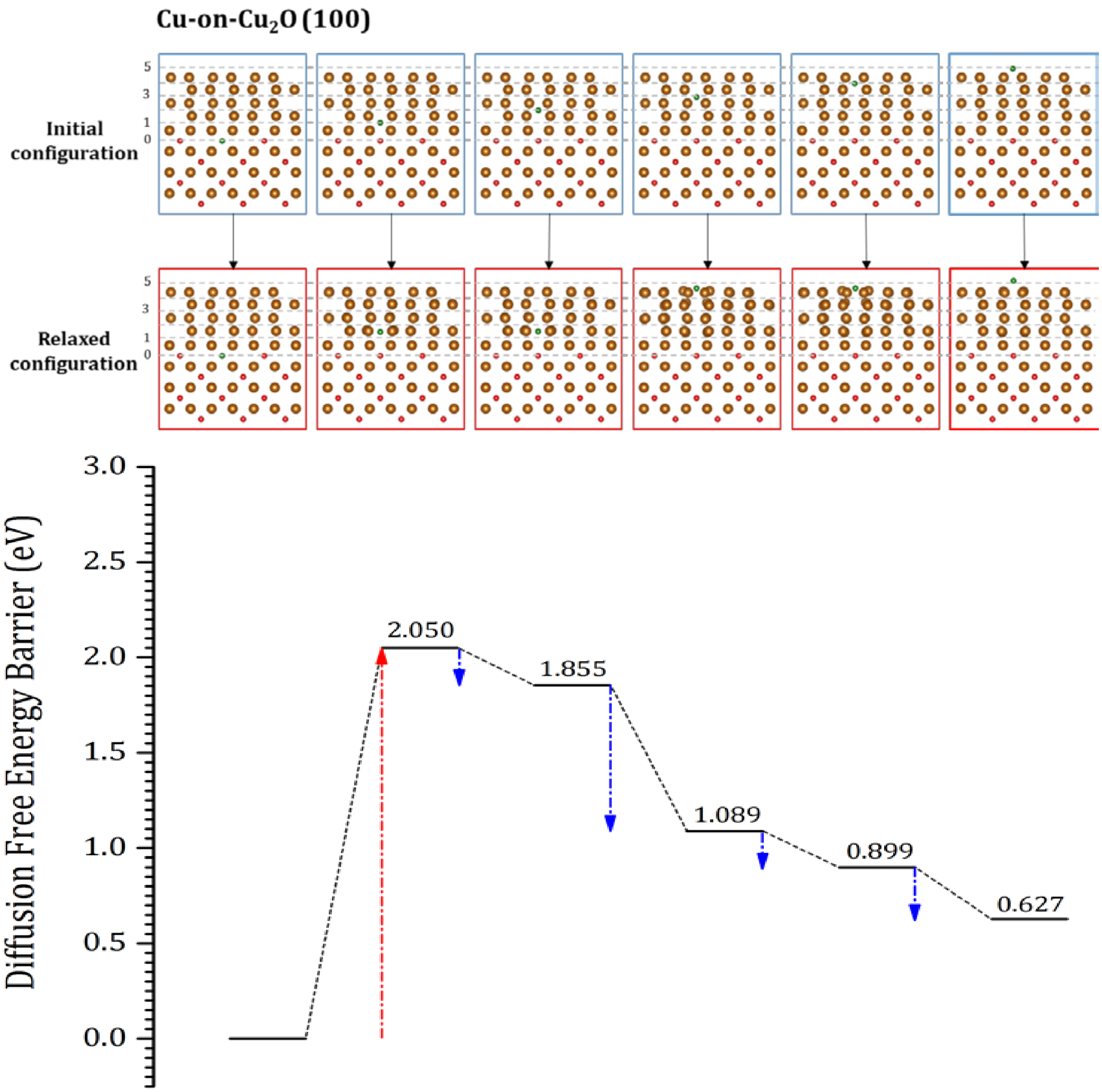

Supplementary Fig. 25. Diffusion-free energy barrier of oxygen atom from (100) facet of Cu-on-Cu$_2$O to the surface. The top panel shows the initial configurations of the displaced oxygen atom at different layers. Bottom panel shows the relevant relaxed configurations. O atom diffuses from the structure (level 0) to the surface (level 5), one level in each step. Brown spheres are copper atoms, while the red ones are oxygen atoms. The green sphere shows the oxygen atom that diffuses. In bottom the diffusion free energy barrier for each step is depicted.

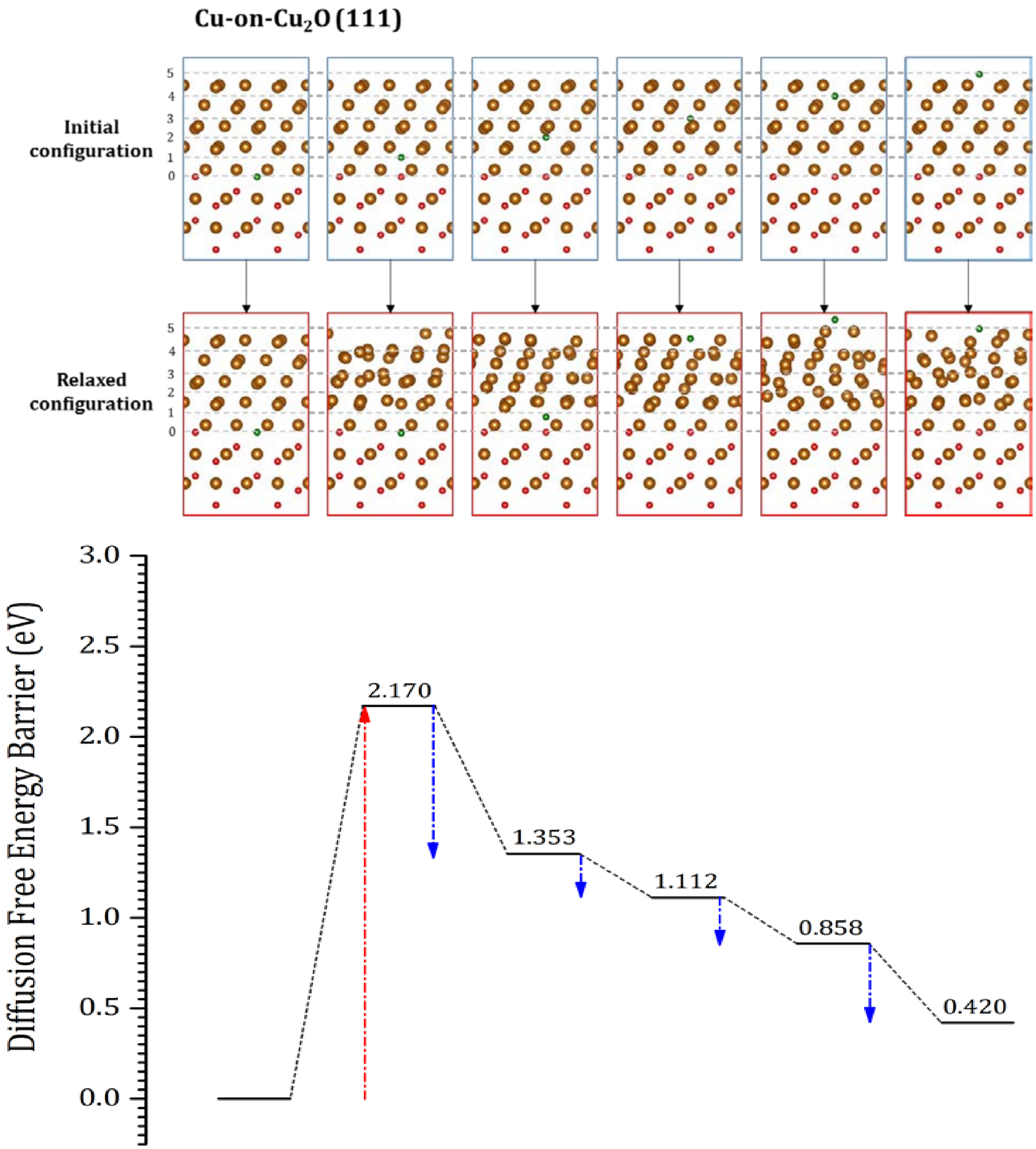

Supplementary Fig. 26. Diffusion free energy barrier of oxygen atom from (111) facet of Cu-on-Cu_2O to the surface. The top panel shows the initial configurations of the displaced oxygen atom at different layers. Bottom panel shows the relevant relaxed configurations. O atom diffuses from the structure (level 0) to the surface (level 5), one level in each step. Brown

spheres are copper atoms, while the red ones are oxygen atoms. The green sphere shows the oxygen atom that diffuses. In bottom the diffusion free energy barrier for each step is depicted.

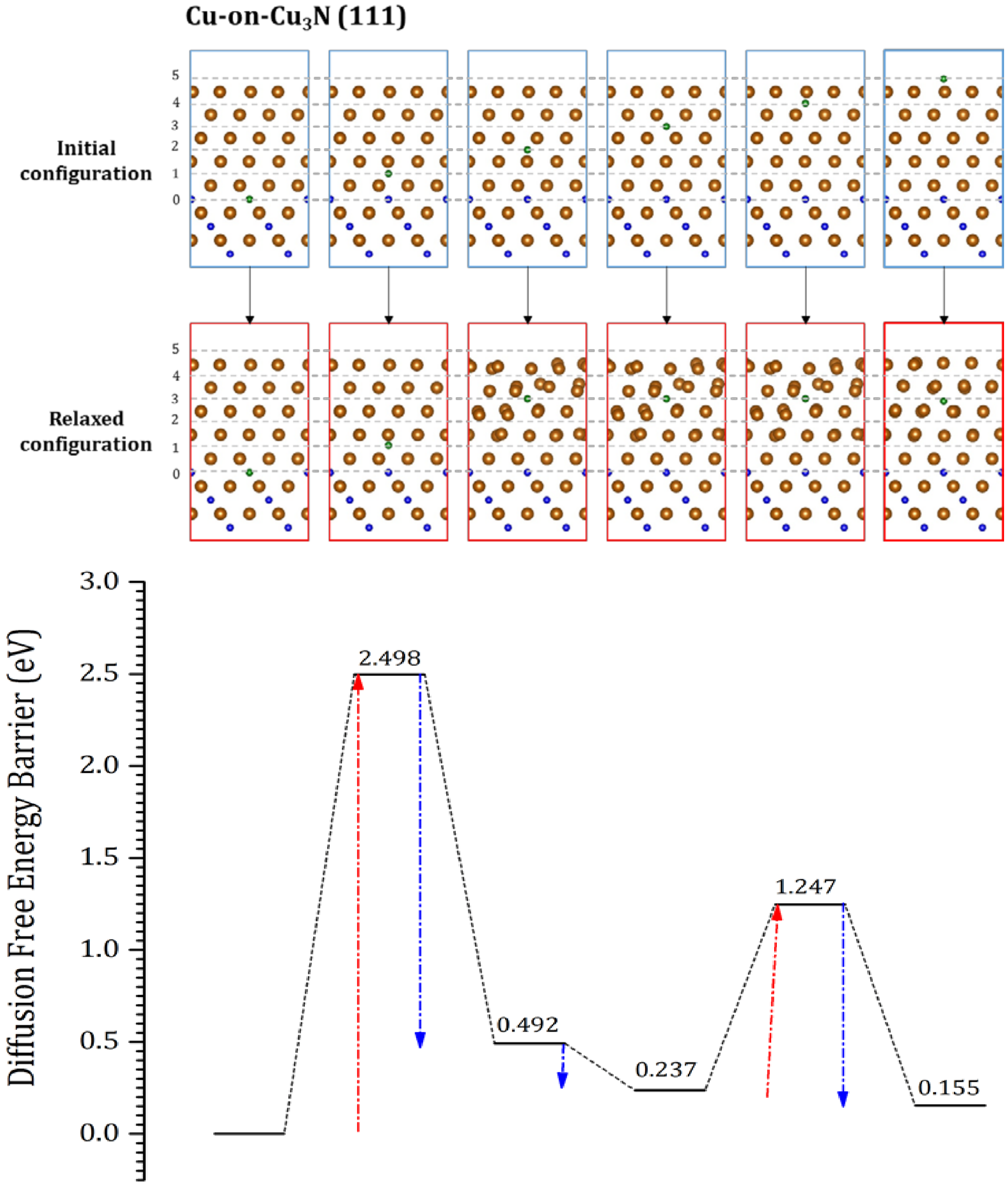

Supplementary Fig. 27. Diffusion free energy barrier of nitrogen atom from (111) facet of Cu-on-Cu$_3$N to the surface. Top panel shows the initial configuration of the displaced nitrogen atom at different layers. Bottom panel shows the relevant relaxed configuration. N atom diffuses from the structure (level 0) to the surface (level 5), one level in each step. Brown spheres are

copper atoms, while the blue ones are nitrogen atoms. The green sphere shows the nitrogen atom that diffuses. In bottom the diffusion free energy barrier for each step is depicted.

Supplementary Table 1. Electrochemical capacitance values and surface roughness factors measured using double-layer capacitance method for the three catalysts.

Electrode	Capacitance	Surface roughness factor	ECSA
Cu-on-Cu_3N	0.28 mF	9.7	1.84 cm^2
Cu-on-Cu_2O	0.23 mF	8.0	1.52 cm^2
Cu	0.27 mF	9.3	1.77 cm^2

Supplementary Table 2. CO_2 reduction Products distribution of at different applied potentials on Cu-on-Cu_3N catalyst.

**Potential (V vs RHE)**	**H_2 (FE, %)**	**CH_4 (FE, %)**	**C_2H_4 (FE, %)**	**HCOOH (FE, %)**	**C_2H_5OH (FE, %)**	**C_3H_7OH (FE, %)**
**-0.55**	64.8±8	2.9±0.1	4.5±0.1	14.4±0.2	0	0
**-0.65**	50.3±3	6.1±0.05	11.3±0.7	13.0±0.5	0	0
**-0.75**	37.0±3	11.5±0.1	27.0±1	12.5±0.5	6.2±0.1	0
**-0.85**	27.9±1	8.9±0.2	35.0±1	9.6±0.5	6.0±0.1	2.8±0.1
**-0.95**	17.0±0.5	2.5±0.1	39.3±3	6.6±0.2	18.4±0.3	6.0±0.2
**-1.05**	17.8±1	3.0±0.05	43.0±2	6.0±0.3	15.1±0.2	4.4±0.05
**-1.15**	25.5±3	3.3±0.1	39.0±1	6.2±0.2	15.0±0.2	2.2±0.1
**-1.25**	29.0±0.5	3.0±0.3	40.9±0.5	3.3±0.2	11.0±0.4	1.4±0.02
**-1.35**	43.0±1	2.8±0.3	36.0±1	2.9±0.2	9.1±0.3	1.9±0.02
**-1.45**	53.1±5	3.8±0.1	28.0±1.5	2.2±0.1	4.8±0.3	0

Supplementary Table 3. CO_2 reduction products distribution at different applied potentials on Cu-on-Cu_2O catalyst.

**Potential (V vs RHE)**	**H_2 (FE, %)**	**CH_4 (FE, %)**	**C_2H_4 (FE, %)**	**HCOOH (FE, %)**	**C_2H_5OH (FE, %)**	**C_3H_7OH (FE, %)**
**-0.55**	63.8±5	1.8±0.05	2.4±0.5	21.0±0.2	0	0
**-0.65**	56.7±3	6.7±0.05	9.0±0.2	16.0±0.4	0	0
**-0.75**	54.3±1	6.3±0.1	12.6±1	14.3±0.5	0	0
**-0.85**	40.3±1	9.7±0.1	19.3±2	12.8±0.2	4.7±0.1	1.6±0.05
**-0.95**	27±1.5	10.3±0.15	27.0±1	10.3±0.05	8.9±0.05	4.0±0.1
**-1.05**	30±0.5	12.7±0.1	23.0±0.5	6.6±0.1	7.3±0.3	3.5±0.05
**-1.15**	32.6±1	10.9±0.2	22.0±1	7.3±0.2	5.4±0.5	3.6±0.02
**-1.25**	45.7±6	8.4±0.1	20.6±2	4.3±0.05	2.8±0.1	2.9±0.05
**-1.35**	54±5	8.9±0.05	18.7±1	2.7±0.2	1.7±0.2	1.7±0.04
**-1.45**	63.1±7.5	9.3±0.1	14.0±0.5	2.2±0.1	2.1±0.5	0

Supplementary Table 4. CO_2 reduction products distribution at different applied potentials on pure Cu catalyst.

Potential (V vs RHE)	H_2 (FE, %)	CH_4 (FE, %)	C_2H_4 (FE, %)	HCOOH (FE, %)	C_2H_5OH (FE, %)	C_3H_7OH (FE, %)
**-0.55**	65.3±2	3.9±0.05	0.8±0.03	13.5±0.7	0	0
**-0.65**	58.3±1.8	12.6±0.1	5.0±0.1	11.5±0.05	0	0
**-0.75**	37.6±0.5	28.6±1	11.3±0.05	12.0±0.3	1.6±0.05	0
**-0.85**	27.3±0.3	39.0±1	13.2±0.2	8.7±0.1	3.6±0.1	0
**-0.95**	9.3±0.5	51.0±1.3	23.0±0.5	6.6±0.1	6.8±0.2	2.9±0.02
**-1.05**	9.0±0.2	53.0±2	26.0±1	5.8±0.15	3.0±0.05	1.3±0.05
**-1.15**	11.9±0.2	52.6±2.4	29.0±1	5.3±0.1	0	0
**-1.25**	24.0±1	49.0±2	19.7±1	5.1±0.03	0	0
**-1.35**	35.7±1.5	43.0±2.4	15.0±0.7	5.5±0.05	0	0
**-1.45**	38.4±1.7	43.0±1	13.7±1	4.3±0.1	0	0

Supplementary Table 5. Average partial oxidation state of the Cu atoms on the top layer of each model (calculated by the Bader charge analysis method), CO adsorption energy, CO dimerization energy barrier, and free energy barriers of CO protonation (minimum barrier between COH and CHO).

Model	Partial oxidation state	CO adsorption energy	CO dimerization energy barrier	Energy barrier for CO protonation
Cu (100)	0.000	-0.976	**0.987**	0.721
Cu (111)	0.000	-0.922	1.430	0.650
Cu_3N (100)	0.470	-1.324	**1.306**	1.216
Cu_3N (111)	0.310	-1.530	1.557	1.199
Cu_2O (100)	0.259	-1.568	**1.593**	0.819
Cu_2O (111)	0.370	-1.263	1.700	0.725
Cu-on-Cu_3N (100) – 1 layer	0.250	-1.230	**0.884**	0.933
Cu-on-Cu_3N (100) – 2 layers	0.012	-1.277	1.021	0.892
Cu-on-Cu_3N (111) – 1 layer	0.005	-1.202	**1.255**	0.856
Cu-on-Cu_3N (111) – 2 layers	0.000	-1.153	1.310	0.824
Cu-on-Cu_3N (111) – 3 layers	0.000	-1.169	1.356	0.895
Cu-on-Cu_3N (111) – 4 layers	0.000	-1.028	1.417	0.893
Cu-on-Cu_2O (100) – 1 layer	0.035	-1.452	**0.926**	0.749
Cu-on-Cu_2O (100) – 2 layers	0.013	-1.380	1.205	0.801
Cu-on-Cu_2O (100) – 3 layers	0.015	-1.377	1.492	0.793
Cu-on-Cu_2O (100) – 4 layers	0.00	-1.401	1.594	0.814
Cu-on-Cu_2O (111) – 1 layer	0.029	-1.116	**1.286**	0.661
Cu-on-Cu_2O (111) – 2 layers	0.000	-1.063	1.412	0.712
Cu-on-Cu_2O (111) – 3 layers	0.000	-1.004	1.428	0.714
Cu-on-Cu_2O (111) – 4 layers	0.000	-0.995	1.409	0.703

Supplementary Table 6. Diffusion free energy barrier of nitrogen and oxygen from bulk Cu-on-Cu_3N and Cu-on-Cu_2O structures to the top layers towards the surface. Different levels (0 to 5) are demonstrated in Figure S24-26.

Model	Diffusion free energy barrier (eV)				
	0 (bulk) to 1	1 to 2	2 to 3	3 to 4	4 to 5 (surface)
Cu-on-Cu_3N (111)	2.498	-2.006	-0.255	1.010	-1.092
Cu-on-Cu_2O (100)	2.050	-0.195	-0.765	-0.190	-0.272
Cu-on-Cu_2O (111)	2.170	-0.817	-0.241	-0.254	-0.438

Supplementary Methods

Computational details. The projected augmented wave (PAW) approach[1, 2] and the generalized gradient approximation (GGA) of Perdew, Burke and Ernzerhof (PBE)[3] - as widely accepted functionals for heterogeneous catalysis calculations – employed in the Vienna *ab initio* Simulation Package (VASP)[4] are used to perform all the plane wave density functional theory (DFT) computations. Five different models with different structures are considered: (1) pristine copper (Cu), (2) copper nitride (Cu_3N), (3) copper oxide (Cu_2O), (4) copper-on-copper nitride (Cu-on-Cu_3N), and (5) copper-on-copper oxide (Cu-on-Cu_2O). For each compound, two crystalline facets of (100) and (111) are chosen to study the CO adsorption, CO protonation and CO dimerization on them. Four different number of Cu layers (1, 2, 3 and 4 layers) are considered on top of Cu_3N and Cu_2O for Cu-on-Cu_3N and Cu-on-Cu_2O models. For (100) facet of Cu-on-Cu_3N model, only 1 and 2 Cu top layers are considered. All these models are demonstrated in Supplementary Fig. 18-22.

Dipole corrections and spin polarization are implemented. To resemble the real bulk material and the surface, the top layers (2 top layers in Cu, Cu_3N and Cu_2O, and all Cu monolayers in Cu-on-Cu_3N and Cu-on-Cu_2O) are free to move due to interaction with the adsorbates, while the other layers are fixed in their optimized crystalline positions. The energy convergence tests showed that a vacuum equal or greater than 20 Å with a cut-off energy for the plane wave basis sets of 450 eV and the $2 \times 2 \times 1$ Γ-centered Monkhorst-Pack mesh for the k-points sampling in the first Brillouin zone with a first order Methfessel-Paxton smearing parameter σ of 0.2 eV is enough to ensure that the accuracy in the energy of the system is more than 1 meV. The self-consistent field (SCF) convergence criterion is set to 1×10^{-4} eV for electronic iteration and the ionic relaxation continued till the maximum force was less than 0.02 eV/Å that was updated by the conjugate gradient approach. For charge density calculations, the k-points grid increased to $4 \times 4 \times 1$, and the Bader charge analysis method is used to calculate the charge density on each ion. The average oxidation state of Cu atoms on top layer of each model is mentioned in Supplementary Table 5.

To calculate the CO adsorption energy we use the following equation:

$$E_{CO}^{ads} = E_{*CO} - [E_{*} + E_{CO}] \quad 1$$

Where, E_{*CO} is the energy of the system including both catalyst surface and the CO adsorbate, E_* is the energy of the catalyst surface, and E_{CO} is referred to the stable molecular form of CO in gas phase. The CO adsorption energy on each model is calculated on three different top, hollow and bridge sites and the more favorable one with the most negative energy is mentioned in Supplementary Table 5.

Different mechanisms for CO_2 reduction to C_{2+} products such as ethylene, ethanol and n-propanol are proposed in literature, but in all of them, C-C coupling is a key energy barrier. Since it has been already determined that CO reduction provides the same product distribution as CO_2 reduction, both experimentally and theoretically, so many mechanisms are based on the assumption that CO_2 is converted to CO before undergoing further reduction to multi-carbon hydrocarbons [5]. Koper group has proposed CO dimerization as the first step towards multi-carbon hydrocarbons production [6]. The same mechanism has been widely used by different groups to evaluate the effect of the catalyst [7], surface coverage and field effect [8]. Upon this idea, Goddard's group [9] built a more complete mechanism considering potential effect on C-C coupling. They showed that for applied potential less negative than -0.6 V vs. RHE (reversible hydrogen electrode), the CO dimerization is the rate determining step, however, for potential less than -0.85 V vs. RHE, C-C coupling proceeds through CO dimerization followed by hydrogenation of an oxygen atom ($^*OCCO + H \rightarrow {}^*OCCOH$). In contrast to this study, Bell and Head-Gordon groups[10, 11] showed that, at high applied potential (more negative than -1 V vs. RHE), $^*CHO$ formation is favorable and follows by a reaction with $^*CO$ to form $^*COCHO$, over CO dimerization and subsequent hydrogenation. Norskov group [12] shows that non-electrochemical CO dimerization can be significantly facilitated by considering a charged water layer above the catalyst surface, to simulate the electrolyte effect on the reaction mechanism. This charged water layer was created by considering a cation in a layer of water. They showed that even considering the electric field cannot decrease the activation energy barrier as much as the charged water layer does. Other non-electrochemical steps such as CH_2 dimerization is also proposed by Janik and Asthagiri groups [13, 14] as a possible pathway for multi-carbon hydrocarbons production.

In this study, however, we just considered the non-electrochemical CO dimerization mechanism to compare the catalytic activity of different proposed models. Therefore, the effect of

parameters such as reaction environment, cations and anions within the electrolyte, and applied potential are neglected and assumed to have similar effects on CO dimerization on all different catalysts' surfaces considered in this study. Therefore, the reported results are valid within these assumptions. To calculate the CO-CO coupling energy barrier, two configurations on different active sites are studied: (1) "cis" configuration, in which C-C bond is parallel to the catalyst surface and both oxygen atoms point outward, and (2) "trans" configuration, in which O-C bond is parallel to the catalyst surface, and O-C-C makes an open angle of c.a. 124°, 135°, 116°, 119° and 125° on Cu, Cu_3N, Cu-on-Cu_3N, Cu_2O and Cu-on-Cu_2O surfaces, respectively. These two configurations are demonstrated in Supplementary Fig. 23. In all surface models, the "trans" configuration with O-C bond parallel to the surface, attained the lowest energy. The CO-CO coupling energy barrier on each model is mentioned in Supplementary Table 5. We observed that increasing the number of top Cu layers decreases the sublayer nitrogen and or oxygen effect on the surface electronic structure, and consequently the CO dimerization energy barrier increases for structures with more than 2 copper top layers.

To compare the activation energy barriers of CO dimerization on Cu-on-Cu_3N and Cu-on-Cu_2O catalysts, the transition state energy is calculated on (100) facet of these models with 1 Cu top layer. Activation energy barrier calculations were performed using the climbing image nudged elastic band method (CI-NEB)[15] with 8 images (IMAGE = 8) and a default spring constant (SPRING = -5). In all CI-NEB computations, the SCF convergence criteria is decreased to 1×10^{-7} eV. The calculations started with IBRION = 3 and a force scaling constant of POTIM = 0.01. Then it switched to the conjugate gradient algorithm (IBRION = 2) and a harsher force constant (POTIM = 0.1). After convergence achieved, the vibrational modes for the transition state (*i.e.* the configuration with the highest energy) were calculated using the central finite difference method with two displacements (NFREE = 2), which is implemented in VASP (IBRION = 5). All vibrational frequencies are real (*i.e.* positive) except one (*i.e.* negative), indicating that the transition state is a reliable first order saddle point. These energy barriers are exhibited in Figure S23. The CO dimerization activation energy barrier difference between Cu-on-Cu_3N (0.935 eV) and Cu-on-Cu_2O (1.114 eV) is c.a. 0.179 eV, while the difference in the thermodynamic free energy barriers on these two catalysts is c.a. 0.042 eV. The difference in the activation energy barrier between Cu-on-Cu_3N and Cu (1.347 eV) is c.a. 0.412 eV. This lower

activation energy barrier for CO dimerization justifies that why Cu-on-Cu_3N catalyst shows better catalytic activity towards C_{2+} products.

To further compare the CO_2 conversion to C_1 products (e.g. CO, CHOOH, and CH_4) and C_{2+} products (e.g. C_2H_4, C_2H_5OH, and C_3H_7OH), free energy barrier for CO protonation is also calculated. Two possible intermediate products for CO protonation are considered as described in the following equations:

$$CO + (H^+ + e^-) \rightarrow COH \qquad 2$$

$$CO + (H^+ + e^-) \rightarrow CHO \qquad 3$$

Calculated energy barriers of CO protonation for all considered models are mentioned in Supplementary Table 5. We observed that CO protonation on Cu-on-Cu_3N catalyst has a larger energy barrier (0.933 eV for the best Cu-on-Cu_3N catalyst) compared to Cu-on-Cu_2O catalyst (0.749 eV).

The free energy of diffusion of oxygen from Cu-on-Cu_2O with 4 Cu top layers to the surface of the slab is also calculated. The energy barrier to diffuse from the Cu_2O structure (level 0 in Figures Supplementary Fig. 25-26) to the first layer (level 1) is very high (2.05 eV for (100) facet and 2.17 eV for (111) facet) compare to the diffusion barrier from the first layer (level 1) to the next layers (level 2 to 5). The same calculation is done for the nitrogen diffusion from Cu-on-Cu_3N with 4 Cu top layers to the surface (Supplementary Fig. 27), and similar trend is observed (2.498 eV for the first diffusion barrier). This is in contrast to the previous observation by Garza *et al.* [16] when there is no thermodynamic energy barrier for oxygen diffusion from sublayer to the surface, with a surmountable activation energy barrier. However, obtaining a comparatively high thermodynamic energy barrier in our calculations is not unexpected, because unlike Garza's model, in our models both oxygen and nitrogen need to move from a pristine crystalline structure (Cu_3N or Cu_2O) and leave a vacancy defect in their original position. In addition this diffusion will create a defect in top layers (which were pure Cu). These defect creation phenomena are typically energy demanding. In Garza's model, however, subsurface oxygen is a defect inside the pristine copper structure and attaining a more negative (favorable) thermodynamic energy after diffusion to surface is reasonable. In our simulations for diffusion of oxygen or nitrogen from the first layer to the next top layers, similar to what Garza *et al.* observed, we do not observe any energy barrier and the process is exergonic. Interestingly, for nitrogen diffusion from the last top

layer to the surface, there is another energy barrier (1.01 eV) which does not exist for oxygen. This means that nitrogen atoms prefer to stay in the sublayer rather than to diffuse to the surface. This energy barrier might lead to nitrogen atoms to be trapped in the sublayer, while oxygen atoms easily diffuse to the surface without any energy barrier at this stage. Based on these results we posit that nitrogen atoms can exist in sublayer very close to the surface, while sublayer oxygen atoms will diffuse to the surface and leave the structure as pure copper. The simulation results are depicted in Supplementary Fig. 25-27, and the diffusion free energy barriers are mentioned in Supplementary Table 6.

Supplementary references

1. Blöchl, P.E. Projector augmented-wave method. *Phys. Rev. B* **50**, 17953 (1994).
2. Kresse, G. & Joubert, D. From ultrasoft pseudopotentials to the projector augmented-wave method. *Phys. Rev. B* **59**, 1758 (1999).
3. Perdew, J.P., Burke, K. & Ernzerhof, M. Generalized gradient approximation made simple. *Phys. Rev. Lett.* **77**, 3865 (1996).
4. Kresse, G. & Furthmüller, J. Efficient iterative schemes for ab initio total-energy calculations using a plane-wave basis set. *Phys. Rev. B* **54**, 11169 (1996).
5. Schouten, K.J.P., Kwon, Y., van der Ham, C.J.M., Qin, Z. & Koper, M.T.M. A new mechanism for the selectivity to C_1 and C_2 species in the electrochemical reduction of carbon dioxide on copper electrodes. *Chem. Sci.* **2**, 1902-1909 (2011).
6. Calle-Vallejo, F. & Koper, M.T.M. Theoretical Considerations on the Electroreduction of CO to C_2 Species on Cu(100) Electrodes. *Angew. Chem. Int. Ed.* **52**, 7282-7285 (2013).
7. Xiao, H., Goddard, W.A., Cheng, T. & Liu, Y. Cu metal embedded in oxidized matrix catalyst to promote CO_2 activation and CO dimerization for electrochemical reduction of CO_2. *Proc. Natl. Acad. Sci.* **114**, 6685-6688 (2017).
8. Sandberg, R.B., Montoya, J.H., Chan, K. & Nørskov, J.K. CO-CO coupling on Cu facets: Coverage, strain and field effects. *Surf. Sci.* **654**, 56-62 (2016).
9. Cheng, T., Xiao, H. & Goddard, W.A. Full atomistic reaction mechanism with kinetics for CO reduction on Cu(100) from ab initio molecular dynamics free-energy calculations at 298 K. *Proc. Natl. Acad. Sci.* **114**, 1795-1800 (2017).
10. Goodpaster, J.D., Bell, A.T. & Head-Gordon, M. Identification of Possible Pathways for C–C Bond Formation during Electrochemical Reduction of CO_2: New Theoretical Insights from an Improved Electrochemical Model. *J. Phys. Chem. Lett.* **7**, 1471-1477 (2016).
11. Garza, A.J., Bell, A.T. & Head-Gordon, M. Mechanism of CO_2 Reduction at Copper Surfaces: Pathways to C_2 Products. *ACS Catal.* **8**, 1490-1499 (2018).
12. Montoya, J.H., Shi, C., Chan, K. & Nørskov, J.K. Theoretical Insights into a CO Dimerization Mechanism in CO_2 Electroreduction. *J. Phys. Chem. Lett.* **6**, 2032-2037 (2015).
13. Nie, X., Luo, W., Janik, M.J. & Asthagiri, A. Reaction mechanisms of CO_2 electrochemical reduction on Cu(1 1 1) determined with density functional theory. *J. Catal.* **312**, 108-122 (2014).
14. Nie, X., Esopi, M.R., Janik, M.J. & Asthagiri, A. Selectivity of CO_2 Reduction on Copper Electrodes: The Role of the Kinetics of Elementary Steps. *Angew. Chem. Int. Ed.* **52**, 2459-2462 (2013).

15. Henkelman, G. & Jónsson, H. Improved tangent estimate in the nudged elastic band method for finding minimum energy paths and saddle points. *J. Chem. Phys.* **113**, 9978-9985 (2000).

16. Garza, A.J., Bell, A.T. & Head-Gordon, M. Is Subsurface Oxygen Necessary for the Electrochemical Reduction of CO_2 on Copper? *J. Phys. Chem. Lett.* **9**, 601-606 (2018).
